# High-velocity gunshot chest trauma: tailoring patient care through insights from a mass casualty experience

**DOI:** 10.1007/s00068-026-03264-8

**Published:** 2026-07-29

**Authors:** Aviel Avraham Azulay, Michael Stein, Limor Yamit Tabo, Abraham Lebenthal, Leonid Ruderman, Yael Refaely

**Affiliations:** 1 Department of Cardiothoracic Surgery, Soroka Medical Center, Ben-Gurion University of the Negev, Beer Sheva, Israel; 2 Medical School for International Health, Ben-Gurion University of the Negev, Beer-Sheva, Israel

**Keywords:** Thoracic trauma, Mass casualty incident, Gunshot wounds, Penetrating chest trauma, High-velocity firearm injury, Pulmonary embolism, Brachial plexus injury, Rib fixation

## Abstract

**Objectives:**

This study describes the management strategies and clinical outcomes of a cohort of patients sustaining high-velocity military rifle gunshot wounds to the thorax during a mass casualty incident (MCI). The analysis focuses on damage control surgical techniques, the incidence and timeline of venous thromboembolism (VTE), regional pain management, and early psychiatric protocols.

**Methods:**

A descriptive retrospective analysis was conducted on patients presenting with a single penetrating high-velocity ballistic wound to the thorax on October 7, 2023. Inclusion criteria were limited to patients with a projectile trajectory traversing the lung parenchyma, managed by the cardiothoracic surgery department. Injuries to immediately adjacent structures (subclavian vessels, brachial plexus, clavicle, and scapula) were recorded as associated injuries. Patients presenting with multi-system trauma involving entirely separate, distant anatomical regions were excluded.

**Results:**

The cohort comprised 11 male patients (age range: 19–49 years; 9 soldiers, 2 civilians) injured by 7.62 mm assault rifle rounds. Six patients (54.5%) required emergency thoracotomy, sternotomy, or VATS within the first 24 h due to ongoing hemorrhage; primary hemorrhage control consisted of deep parenchymal lung suturing (*n* = 5) and temporary chest packing (*n* = 1). Four patients (36.4%) underwent delayed, elective surgical stabilization of rib fractures. All patients survived to discharge and were successfully weaned from mechanical ventilation, with no cases of ARDS, pneumonia, or oxygen requirement at discharge. Venous thromboembolism occurred in 45.5% (*n* = 5), including deep vein thrombosis (*n* = 3) and pulmonary embolism (*n* = 2). Ipsilateral brachial plexus injury was present in 63.6% (*n* = 7), occurring exclusively in patients wearing ballistic protective vests. At 3-month follow-up, 4 patients had persistent upper limb neuropathy and 1 reported chronic post-thoracotomy pain. Mean hospital length of stay was 15.27 days (range: 2–41).

**Conclusions:**

Lung-preserving, suture-based damage control strategies were effective for hemorrhage control during this mass casualty surge and supported rapid operative management in a resource-constrained setting. A high incidence of VTE (45.5%) was observed, including pulmonary embolism without documented lower-extremity deep vein thrombosis, supporting the need for early post-stabilization diagnostic surveillance. Brachial plexus injury was common among patients wearing ballistic protection and was associated with long-term functional impairment in this cohort.

## Introduction

Penetrating gunshot wounds (GSW) to the thorax are a major component of emergency and military trauma medicine, posing profound clinical challenges when inflicted by high-velocity military assault rifles. Globally, firearm-related trauma remains a critical public health crisis, causing an estimated 250,000 deaths annually [1]. While substantial literature exists on low-velocity or general penetrating chest trauma [2], detailed data focusing specifically on high-velocity military rifle injuries traversing the lung parenchyma remain limited.

High-velocity projectiles (e.g., 7.62 mm rounds) transfer large amounts of kinetic energy to tissue, producing cavitation, secondary blast effects, and extensive fragmentation [3, 4]. These mechanisms result in complex, non-linear wound tracts that differ substantially from low-velocity injuries and limit the applicability of standard linear surgical techniques such as pulmonary tractotomy, thereby complicating hemorrhage control.

Beyond initial surgical management, high-velocity thoracic trauma is associated with significant systemic complications. Venous thromboembolism (VTE), including deep vein thrombosis and pulmonary embolism, is a recognized contributor to morbidity in severely injured patients [5, 6]. In this context, pulmonary endothelial injury may contribute to early in-situ thrombosis independent of peripheral deep vein thrombosis [7, 8], while optimal timing of prophylaxis remains challenging due to concurrent bleeding risk.

Chest wall injury management and pain control are also critical determinants of outcome. Surgical stabilization of rib fractures (SSRF) is well established in blunt thoracic trauma [9, 10], but its role in penetrating ballistic injury remains less defined due to the nature of comminuted fractures and associated soft-tissue disruption [11]. In parallel, acute psychological trauma following firearm injury requires early structured intervention to mitigate the risk of post-traumatic stress disorder (PTSD) [12, 13], though standardized non-benzodiazepine approaches in disaster settings remain incompletely described.

The management of such injuries is further complicated during mass casualty incidents (MCI). On October 7, 2023, Soroka University Medical Center, a regional Level 1 trauma center in southern Israel, managed an unprecedented Level 3 MCI involving 680 casualties within 24 h, with peak arrival rates exceeding 83 patients per hour [14]. This surge required full hospital mobilization and reorganization of operative trauma pathways, with the cardiothoracic surgery service assuming centralized responsibility for penetrating thoracic injuries to optimize operative throughput and resource allocation.

This study presents a descriptive, retrospective analysis of a cohort of 11 patients presenting with high-velocity military rifle wounds traversing the thoracic cavity, managed entirely during this single-day mass casualty surge. We describe operative strategies, damage control approaches, venous thromboembolism patterns, chest wall management, and early psychiatric interventions in this unique setting. We aim to provide hypothesis-generating data to inform future development of standardized management approaches for high-velocity thoracic trauma in mass casualty settings.

## Methods

### Ethics and regulatory approval

This study was conducted in accordance with the Declaration of Helsinki. Ethical approval was obtained from the Institutional Review Board and Helsinki Ethics Committee of Soroka University Medical Center. The requirement for informed consent was waived due to the retrospective design and anonymized data extraction from electronic medical records.

### Study design and timeline

This study is a single-center, retrospective observational analysis conducted at Soroka University Medical Center, a designated regional Level 1 trauma center. The cohort includes consecutive patients presenting with penetrating high-velocity ballistic chest trauma during a Level 3 mass casualty incident on October 7, 2023. All admissions occurred during the same-day surge period. The study period extended to January 1, 2024, to allow for standardized 3-month follow-up. Reporting follows STROBE (Strengthening the Reporting of Observational Studies in Epidemiology) guidelines [15]. 

### Patient selection and criteria

#### Inclusion criteria

Patients were included if they sustained a single high-velocity gunshot wound to the thorax from a military assault rifle (7.62 mm), with projectile trajectory traversing the lung parenchyma, and were managed by the cardiothoracic surgery service. Injuries to adjacent structures (subclavian vessels, brachial plexus, clavicle, scapula) were recorded as associated injuries but did not exclude inclusion.

#### Exclusion criteria

Patients with multi-system trauma involving anatomically distant injuries (abdominal, cranial, or major peripheral orthopedic trauma) were excluded to maintain a homogeneous cohort of isolated thoracic ballistic injury.

### Variables and data collection

Clinical data were extracted from institutional electronic medical records. Baseline variables included age, sex, and military/civilian status. Pre-hospital variables included transit time and administration of tranexamic acid (TXA) or dried plasma.

Operative variables included surgical approach (anterolateral thoracotomy, median sternotomy, VATS), damage control strategy (lung suturing vs. chest packing), PRBC transfusion within 12 h, delayed surgical stabilization of rib fractures (SSRF), and regional analgesia (erector spinae plane block vs. epidural).

### Outcome definitions

#### Venous Thromboembolism (VTE)

VTE was defined as radiologically confirmed deep vein thrombosis (DVT) or pulmonary embolism (PE). PE was diagnosed using trauma-protocol contrast-enhanced CT, and DVT via lower-extremity duplex ultrasound performed by hospital day 5. Imaging was performed as early as clinically feasible within 24–48 h of stabilization. In-situ pulmonary artery thrombosis was defined as PE identified without concurrent lower-extremity DVT on systematic evaluation.

#### Clinical sepsis

Sepsis was defined retrospectively as suspected or confirmed infection requiring intravenous antibiotic therapy in the presence of SIRS criteria, including temperature > 38 °C or < 36 °C, heart rate > 90 bpm, respiratory rate > 20/min, or leukocyte abnormalities.

#### Pulmonary recovery metrics

Outcomes included duration of mechanical ventilation, incidence of pneumonia or ARDS, and oxygen requirement at discharge.

#### Follow-up outcomes

Long-term outcomes were assessed at 3 months post-injury and included survival, persistent upper limb neuropathy, chronic post-thoracotomy pain, and return to baseline physical function.

## Results

### Baseline cohort characteristics

The study cohort comprised 11 male patients with a mean age of 26.8 years (range: 19–49). Most were active-duty military personnel (*n* = 9, 81.8%), with the remainder civilians (*n* = 2, 18.2%). All sustained high-velocity penetrating thoracic injuries from 7.62 mm assault rifle rounds. Mean pre-hospital transit time was 185 min (range: 110–310). Field resuscitation included dried plasma in 7 patients (63.6%) and tranexamic acid (TXA) in 10 (90.9%) (Table 1).

**Table 1:** Baseline characteristics and initial management of patients with high-velocity penetrating thoracic trauma (*N* = 11)

Category	Variable	Value
Demographics	Age, years, mean (range)	26.8 (19–49)
	Sex, male, n/N (%)	11/11 (100.0%)
	Military personnel	9/11 (81.8%)
	Civilians	2/11 (18.2%)
Pre-Hospital Phase	Transit time, min, mean (range)	185 (110–310)
	Field tranexamic acid (TXA)	10/11 (90.9%)
	Field dried plasma	7/11 (63.6%)
Initial resuscitation	Tube thoracostomy performed	11/11 (100%)
Early operative need (≤ 12–24 h)	Emergency surgery required	6/11 (54.5%)
Early transfusion	Packed RBC transfusion within the first 12 h (units), mean (range)	2.27 units (0–5)

### Operative interventions and acute management

Patients who had not received pre-hospital tube thoracostomy underwent chest tube insertion on arrival as part of initial resuscitative management in the trauma bay. Four patients (36.4%) required immediate operative intervention due to hemorrhage, including median sternotomy (*n* = 2, 18.2%) and anterolateral thoracotomy (*n* = 2, 18.2%). The remaining 7 patients (63.6%) were initially managed in the ICU following chest tube placement. Of these, 1 patient required subsequent emergent thoracotomy for ongoing hemorrhage. Another underwent VATS for for delayed ongoing intrathoracic bleeding and hemothorax evacuation. (*n* = 1, 9.1%). The remaining 5 patients (45.5%) were managed non-operatively for pulmonary injuries.

Mean PRBC transfusion within the first 12 h was 2.27 units per patient (range: 0–5). Delayed surgical stabilization of rib fractures (SSRF) was performed in 4 patients (36.4%) at a mean of 4.5 days post-injury. Regional analgesia included erector spinae plane blocks in 4 patients (36.4%) and thoracic epidural analgesia in 3 patients (27.3%).

### Associated injuries and wound outcomes

Ipsilateral brachial plexus injury was present in 7 patients (63.6%). All patients sustained complex exit wounds with soft-tissue cavitation. Wound management included primary closure (*n* = 3, 27.3%), vacuum-assisted closure (VAC) therapy (*n* = 3, 27.3%), ribbon dressings with irrigation (*n* = 3, 27.3%), and simple dressings (*n* = 2, 18.2%). All wounds demonstrated progression toward healing by discharge. No delayed bleeding or secondary hemothorax occurred.

### Systemic complications and VTE

VTE occurred in 5 patients (45.5%), including pulmonary embolism in 2 (18.2%) and deep vein thrombosis in 3 (27.3%). Pulmonary embolism was identified on contrast-enhanced CT within 24–48 h of stabilization. DVT was detected by routine lower-extremity ultrasound by hospital day 5. Both PE cases were isolated pulmonary artery thromboses without concomitant DVT. Clinical sepsis occurred in 5 patients (45.5%). Additional complications included localized wound infection (*n* = 1, 9.1%) and transient lung-abscess fistula (*n* = 1, 9.1%).

### Pulmonary recovery and follow-up

Mean hospital length of stay was 15.27 days (range: 2–41). All patients were successfully weaned from mechanical ventilation (mean duration: 1.81 days; range: 0–6). No patient developed pneumonia or ARDS. All patients were discharged on room air. At 3-month follow-up, survival was 100% (*n* = 11). Persistent upper limb neuropathy occurred in 4 patients (36.4%), and chronic post-thoracotomy pain in 1 patient (9.1%) (Table 2).

**Table 2:** Operative interventions, clinical complications, and outcomes

Category	Variable	Value
Surgical approach	Anterolateral thoracotomy	3/11 (27.3%)
	Median sternotomy	2/11 (18.2%)
	Video-assisted thoracoscopic surgery (VATS)	1/11 (9.1%)
Pulmonary Hemorrhage Control	Lung suturing (primary hemostasis)	5/11 (45.5%)
	Temporary chest packing	1/11 (9.1%)
Chest wall management	Delayed SSRF performed	4/11 (36.4%)
Regional analgesia	ESP block	4/11 (36.4%)
	Thoracic epidural	3/11 (27.3%)
Complications	VTE (total)	5/11 (45.5%)
	- DVT - PE	- 3/11 (27.3%) - 2/11 (18.2%)
	Clinical sepsis	5/11 (45.5%)
	Local wound infection	1/11 (9.1%)
	Pulmonary–chest wall fistula	1/11 (9.1%)
Associated injuries	Ipsilateral brachial plexus injury	7/11 (63.6%)
Pulmonary outcomes	Mechanical ventilation duration (days)	1.81 (0–6)
	Oxygen independence at discharge	11/11 (100%)
	Hospital length of stay (days)	15.27 (2–41)
Follow-up (3 months)	Survival	11/11 (100%)
	Persistent brachial plexus neuropathy	4/11 (36.4%)
	Chronic post-thoracotomy pain	1/11 (9.1%)

## Discussion

### Surgical hemostasis and damage control

**Fig. 1:**
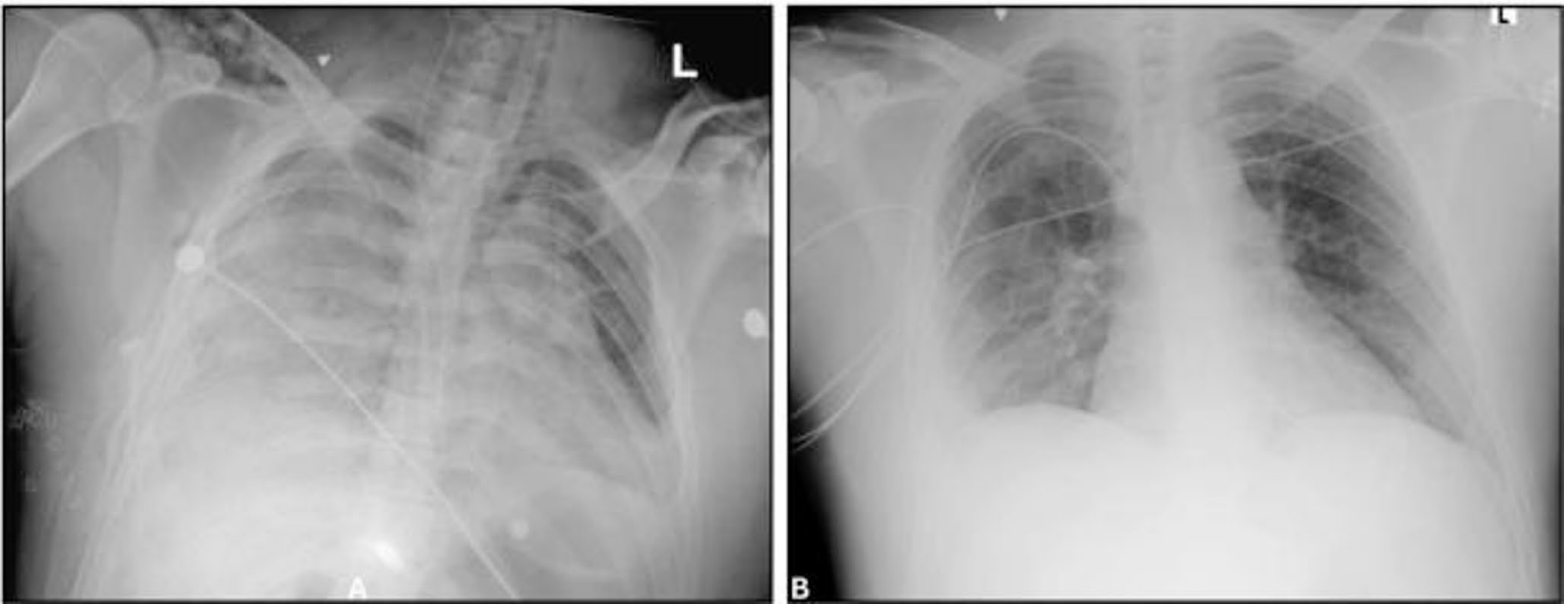
Chest X-rays of a patient with a gunshot injury to the right hemithorax. **A – Postoperative Day (POD) 0**: Extensive pulmonary contusion, multiple rib fractures, subcutaneous emphysema extending into the neck, and visible retained ballistic fragments. A projectile is seen projected over the thoracic spine, located subcutaneously in the posterior thorax. **B – POD 73**: Following surgical extraction of retained bullets from the mandible and subcutaneous tissues of the back, near-complete radiographic resolution of the pulmonary injury is demonstrated

The primary operative objective in high-velocity penetrating lung trauma is rapid hemorrhage control while minimizing operative time to optimize theater throughput during a mass casualty incident (MCI). In this cohort, five of the six operatively managed patients were treated using a lung-preserving suture-only approach. Projectiles fired from military rifles, such as 7.62 mm caliber rounds, transfer high kinetic energy to biological tissues, causing temporary cavitation, secondary blast waves, and projectile or bone fragmentation [3]. These non-linear wound tracts limit the utility of standard linear pulmonary tractotomy techniques and complicate definitive hemorrhage control. Formal anatomical pulmonary resections, including lobectomies, were not performed in this cohort, as they are time-consuming especially during an MCI response. Additionally, a lobectomy imposes functional risks in this population, as high-velocity ballistic chest trauma may cause concurrent contralateral pulmonary contusions due to the trans-thoracic shockwave. Conserving maximal parenchymal volume on the injured side is therefore essential to maintain adequate respiratory reserve. (Fig. 1)

Temporary intra-thoracic packing was required in one patient with persistent diffuse parenchymal and chest wall bleeding in the setting of traumatic coagulopathy. Consistent with damage control principles, temporary closure and delayed re-exploration allowed for correction of coagulopathy, hypothermia, and acidosis prior to definitive closure.

### Surgical access and protective armor limitations

**Fig. 2:**
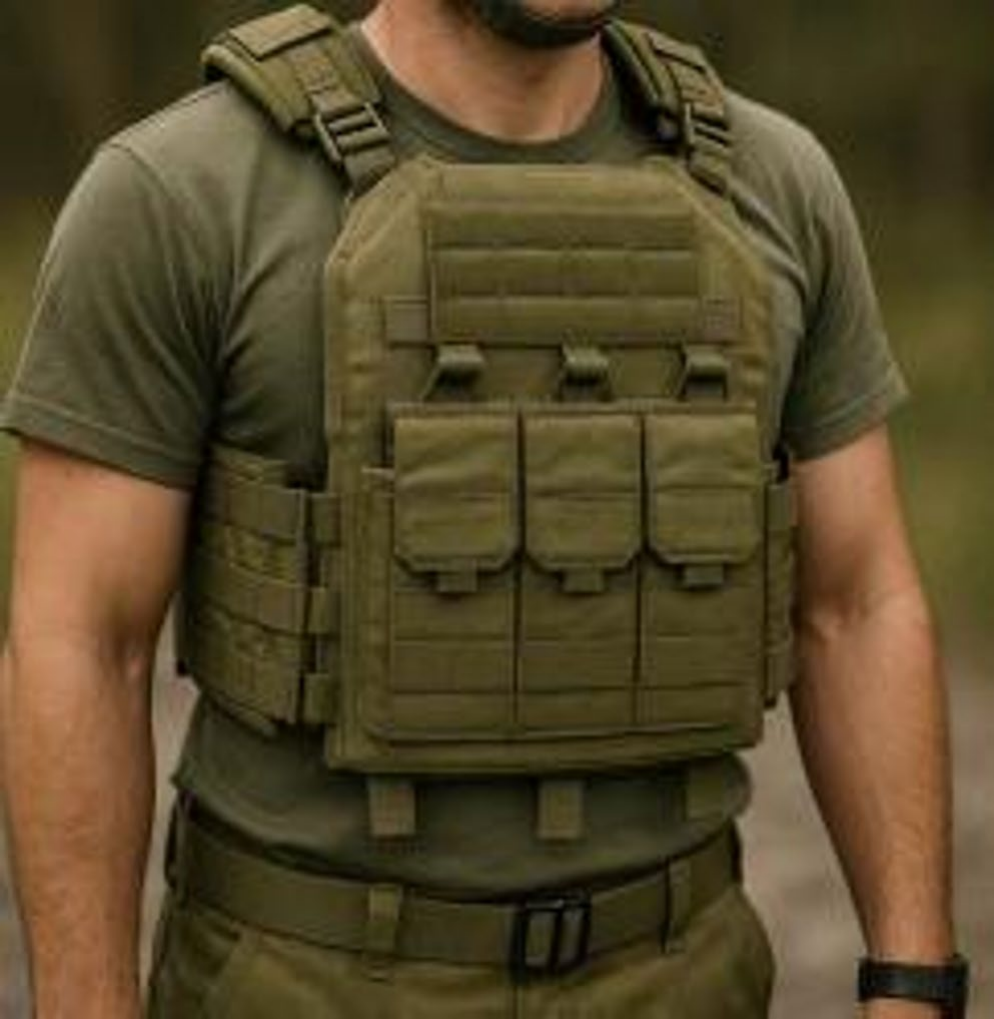
AI-generated illustration of a soldier wearing a protective vest. Note that only the front and back plates are bullet-resistant, leaving the lateral chest, shoulders, and thoracic inlet exposed and vulnerable to injury

The selection of surgical exposure in penetrating thoracic trauma requires balancing rapid access with adequate visualization of potential injury trajectories. While anterolateral thoracotomy remains the standard approach for unilateral chest trauma, median sternotomy was required in two patients in this cohort. Both patients were military personnel wearing standard ceramic ballistic protective vests. (Fig. 2) Contemporary body armor provides effective protection of the central mediastinum and cardiac structures but leaves the lateral thorax, shoulder girdle, and thoracic inlet relatively exposed. In both cases, the projectile trajectory involved the thoracic inlet, raising concern for potential injury to the great vessels and subclavian system.

Median sternotomy was therefore selected to enable rapid proximal vascular control of the innominate and subclavian vessels, which may not be adequately accessible through an anterolateral thoracotomy in this injury pattern. This highlights the importance of integrating projectile trajectory assessment with an understanding of armor coverage zones during initial surgical decision-making.

### Venous thromboembolism: incidence and contributing factors

The overall incidence of VTE in this cohort was 45.5% (*n* = 5/11), occurring despite chemoprophylaxis initiated as early as clinically feasible [5]. In the setting of high-velocity penetrating trauma, clinical management requires balancing hemorrhagic risk against the development of a post-traumatic hypercoagulable state. Universal trauma-protocol CT imaging performed within 24–48 h of admission identified pulmonary embolism in two patients, in the absence of concurrent lower-extremity deep vein thrombosis. This pattern suggests that these early events may represent localized in-situ pulmonary arterial thrombosis rather than classical embolic disease originating from peripheral venous thrombi [7, 8].

Several context-specific factors may have contributed to the observed thrombotic burden. Prolonged pre-hospital evacuation times (mean: 185 min), driven by operational constraints, likely prolonged exposure to shock and tissue ischemia [6]. Additionally, early resuscitation strategies included the administration of tranexamic acid (90.9%) and field-dried plasma (63.6%), both of which may contribute to a prothrombotic milieu in susceptible patients [16]. A substantial proportion of patients (45.5%) also developed clinical sepsis, which is associated with endothelial dysfunction and activation of systemic coagulation pathways [6].

**Fig. 3:**
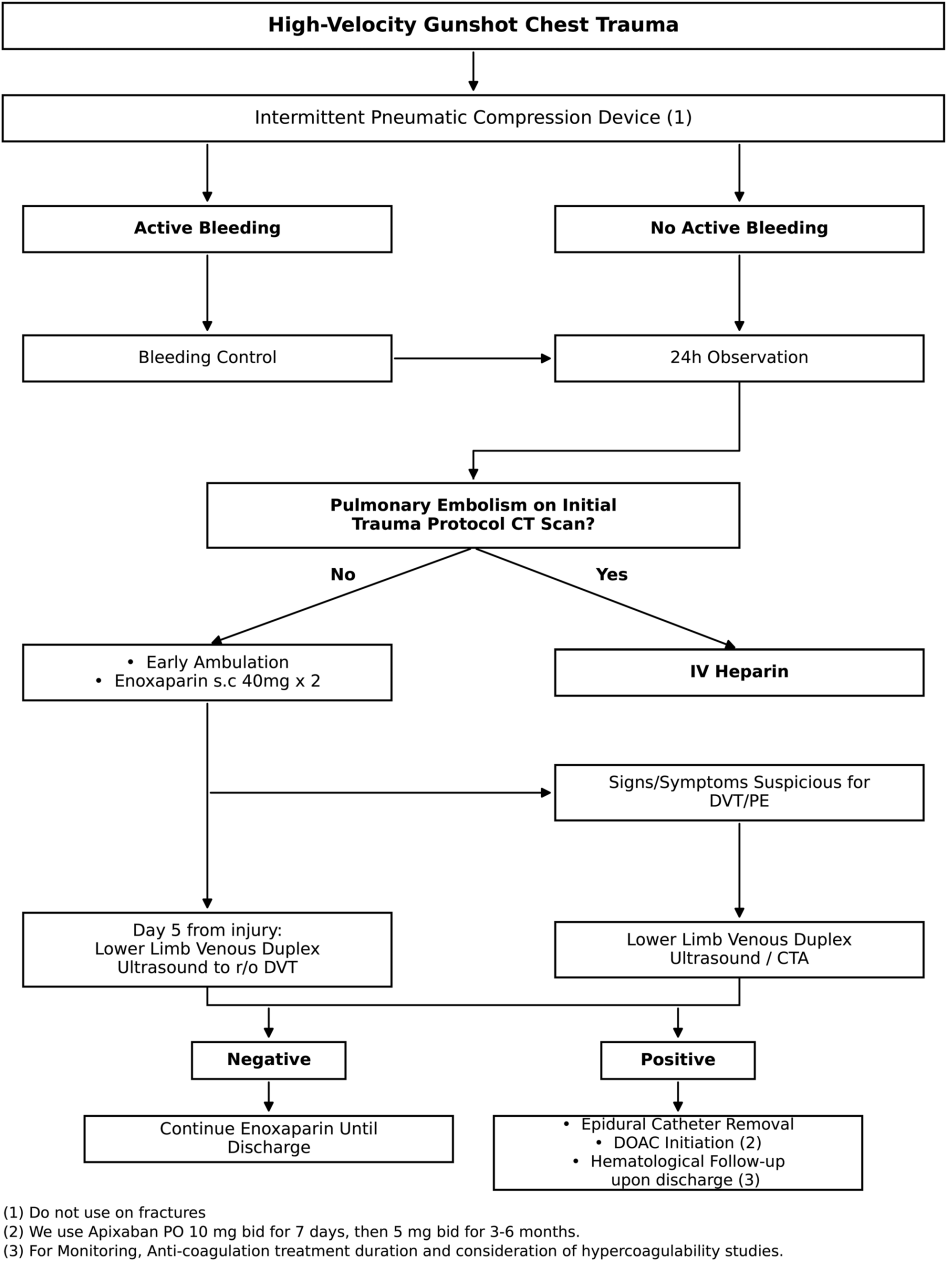
Institutional venous thromboembolism screening and prophylaxis protocol in high-velocity thoracic trauma

These findings highlight the complexity of VTE risk stratification in mass casualty environments and are summarized within the institutional prophylaxis and screening pathway (Fig. 3).

### Surgical stabilization of rib fractures

Surgical stabilization of rib fractures (SSRF) has been shown to reduce ventilator duration and pain in severe blunt thoracic trauma [9, 10], but its role in penetrating high-velocity ballistic injury remains less well defined. In this context, high-velocity exit wounds frequently produce comminuted rib fractures with extensive osseous fragmentation and surrounding soft-tissue injury, resembling a functional flail chest pattern [11].

During this acute 24-hour mass casualty surge, SSRF was deferred to prioritize operative capacity and resource allocation. Fixation was subsequently performed in an elective, delayed fashion (mean 4.5 days post-injury) following hemodynamic stabilization, correction of coagulopathy, and clarification of indications, including refractory chest wall pain with impaired respiratory mechanics or failure to wean from mechanical ventilation [11]. In this context, delayed chest wall stabilization may represent a reasonable operational strategy in disaster and surge settings.

### Exit-wound management

All 11 patients sustained complex exit wounds characterized by soft-tissue cavitation and skin defects [4]. Management strategies included primary closure (*n* = 3), vacuum-assisted closure (VAC) therapy (*n* = 3), deep wound tract ribbon dressings with daily irrigations (*n* = 3), and simple local irrigation with dressing changes (*n* = 2). All approaches demonstrated similar healing progression by the time of discharge, with a low incidence of infectious complications.

In mass casualty settings with constrained resources, the use of less resource-intensive approaches, such as local irrigation and dressing-based management, may be a pragmatic operation choice that preserves personnel and material resources without compromising wound outcomes.

### Regional pain management

Multimodal regional analgesia was used across both operative and non-operative patients in this cohort, with continuous erector spinae plane (ESP) blocks performed in four patients and thoracic epidural analgesia in three patients [17]. Effective pain control is essential in high-velocity chest trauma to mitigate splinting, maximize diaphragmatic excursion, and prevent secondary pulmonary complications such as atelectasis and pneumonia [18].

In non-operative ICU patients, the use of regional anesthesia was associated with adequate pain control and facilitated early mobilization and respiratory stability during the acute recovery phase, suggesting a potential role for regional techniques as part of conservative thoracic trauma management strategies.

### Associated brachial plexus trauma

Ipsilateral brachial plexus injuries were identified in 63.6% (*n* = 7) of patients and were observed exclusively in patients wearing ballistic vest [19]. Injury patterns appeared to correlate with potential geometric gaps in protective coverage, although the exact biomechanical mechanisms are likely multifactorial. In a subset of patients, associated comminuted fractures of the clavicle and scapula may have contributed to nerve injury through secondary osseous fragmentation. In other cases, neurological deficits may reflect indirect mechanisms, including high-energy transfer effects, transient cavitation-related tissue displacement, peri-neural edema, and possible ischemic injury secondary to adjacent vascular trauma [19]. Given the difficulty in differentiating between direct nerve disruption and transient neuropraxia in the acute phase, early multidisciplinary neurological assessment may be warranted to guide the timing of further diagnostic evaluation and to inform decisions regarding conservative management versus delayed surgical exploration.

### Early psychiatric management protocols

Surviving high-velocity ballistic trauma in the setting of a mass casualty incident carries a substantial risk of acute psychological distress and subsequent post-traumatic stress disorder (PTSD) [12, 13]. In this cohort, early psychological management reflected a standardized institutional disaster protocol. The protocol minimized the use of benzodiazepines during the acute phase in accordance with World Health Organization disaster management guidance [20], reflecting concerns that benzodiazepines may interfere with acute stress processing and may be associated with adverse longer-term psychological outcomes, although available evidence remains heterogeneous [21, 22]. Instead, early management incorporated non-benzodiazepine pharmacological support alongside bedside psychiatric assessment. Mirtazapine was used in selected patients to address acute sleep disturbance and hyperarousal. In addition, Prothipendyl (prothipendyl hydrochloride), a sedative-neuroleptic used in psychiatric emergency care settings, was administered in selected cases as an alternative anxiolytic agent.

### Lessons learned from a mass casualty experience

Beyond the clinical findings, this experience provided several practical lessons regarding the organization of care for isolated penetrating thoracic injuries during a mass casualty incident. At our institution, the cardiothoracic surgery service assumed primary responsibility for these patients, allowing the trauma surgeons to remain available for casualties with complex multisystem injuries. During periods of peak patient influx, care was frequently delivered by collaborative trauma and cardiothoracic teams according to personnel availability. The involvement of cardiothoracic surgeons in the management of isolated thoracic ballistic injuries helped preserve trauma surgery capacity for patients with multisystem injuries while providing specialized thoracic surgical expertise. This approach appeared to facilitate efficient resource utilization during this mass casualty response.

Similarly, the management of isolated thoracic trauma patients within a dedicated cardiothoracic intensive care unit proved feasible throughout this MCI. These units routinely manage thoracic surgical patients, chest drains, postoperative bleeding, and respiratory complications, thereby enabling general trauma ICU resources to remain available for patients with multisystem injuries.

While these observations were not formally evaluated as study outcomes, they may provide practical considerations for disaster preparedness planning and future mass casualty response protocols in centers with available cardiothoracic surgical and critical care resources.

### Limitations

This study has several limitations. As a retrospective, single-center analysis of a small cohort (*n* = 11) managed during a specific mass casualty incident, the findings may not be generalizable to other trauma systems or non–mass casualty settings. The highly selected nature of the cohort, restricted to isolated high-velocity penetrating thoracic injuries, further limits external applicability. In addition, management decisions were influenced by real-time operational constraints, including fluctuating resource availability and operating room capacity during the surge period, which may have introduced variability in timing and treatment pathways. The relatively short three-month follow-up period may not fully capture the long-term functional and neurological outcomes, particularly in patients with brachial plexus injury or chronic post-thoracotomy pain. Further prospective, multicenter studies with extended follow-up are warranted to better define long-term outcomes in this injury pattern. In addition, anti-factor Xa monitoring was not performed, limiting precise assessment of the adequacy of pharmacologic venous thromboembolism prophylaxis.

## Conclusion

This study describes the management and outcomes of high-velocity penetrating thoracic injuries treated during a mass casualty incident. Lung-preserving, suture-based surgical strategies were associated with effective hemorrhage control under surge conditions and may support efficient operative throughput in resource-constrained settings. A high incidence of venous thromboembolism was observed, including cases of pulmonary arterial thrombosis without lower-extremity deep vein thrombosis, highlighting the need for heightened diagnostic vigilance in this population. In addition, brachial plexus injury was common among patients wearing ballistic protection and contributed significantly to long-term functional impairment. These findings represent a small, single-center experience and may contribute to the evolving understanding of high-velocity thoracic trauma management during mass casualty incidents.

## Data Availability

No datasets were generated or analysed during the current study.
